# Block Copolymer Membranes—Progress and Challenges

**DOI:** 10.3390/membranes12040354

**Published:** 2022-03-24

**Authors:** Juliana Isabel Clodt

**Affiliations:** Helmholtz-Zentrum Hereon, Institute of Membrane Research, Max-Planck-Str. 1, 21502 Geesthacht, Germany; juliana.clodt@hereon.de

Block copolymers are capable of providing more than one advantageous property due to their selected repeating units, which make them an outstanding candidate for polymer-based membranes. Since “Asymmetric Superstructure Formed in a Block Copolymer via Phase Separation” was published in 2007 [1], a new field has moved into the focus of polymer and membrane scientists. Extrapolating block copolymers and their versatile self-assembly, various block copolymer membranes have gained growing importance with many promising applications in the last decade, including micro-, ultra-, and nanofiltration; cell separation; controlled drug delivery and optics; and gas separation. The Special Issue titled “Block Copolymer Membranes—Progress and Challenges” in the journal *Membranes* aims to emphasize how broad the development of block copolymer membranes is. The discussed topics include the structural formation of flat sheet membranes under various conditions, upscaling opportunities, and the usage of block copolymer template-directed catalytic systems. 

Sankhala et al. report a modification of the fabrication process of isoporous flat sheet membranes conventionally carried out via the evaporation-induced self-assembly of block copolymers in combination with non-solvent induced phase separation (SNIPS). As a new concept, a controlled evaporation is provided using a defined gas flow, and the process is introduced as gSNIPS (gas-flow-induced self-assembly of block copolymers in combination with non-solvent induced phase separation is introduced). Based on a systematic investigation of the gSNIPS concept, the window of parameters for the desired structure formation of the membranes was extended and the structure formation was accelerated. The concentration of the block copolymer can be decreased when the gSNIPS method is used, which will lower the costs of fabrication. Furthermore, the new method has a strong effect on the membrane substructure while the pore sizes remain constant, which will be interesting for performance studies [2]. 

Bucher et al. investigated the upscaling of block copolymer membranes using spraying techniques as a proof of concept. The concentration of block copolymers and consequently the amount of polymer and costs that are necessary for a particular area is strongly decreased by spraying the polymer compared to the commonly used doctor-blade technique. To upscale the spray coating process, three different approaches were pursued, namely air-brush, one-fluid nozzles, and two-fluid nozzles, which are generally used in the coating industry. The different spraying systems were successfully implemented in a membrane casting machine bearing a non-solvent precipitation to allow the phase-separation process. The system was tested using a of polystyrene-*block*-poly(4-vinylpyridine) diblock copolymer solution. The authors provide a detailed examination of the spray pattern and a discussion of limitations of the spraying technique itself. Two-fluid nozzles were identified to be the most promising. Using these nozzles allowed very thin films of around 150 nm thickness and a homogeneous surface to be obtained that exhibited isoporosity. As a limitation, it was found that the low-concentration polymer solution penetrated the support membrane, leading to pore blocking. The permeances were measured to be lower than 50 L/(m^2^hbar), and some membranes were completely blocked. It was identified that the use of fillers, other support materials, or increased viscosity was helpful [3]. 

Kumar et al. summarized the advances of block copolymer self-assembled structures in the field of template-assisted nano-catalysts. The review focuses on the usage of diblock, triblock, and other types of block copolymers studied in this research area, including tuneable periodicity, domain orientation, and degree of lateral orders of self-assembled block copolymers. With these approaches, catalysts can be fabricated and applied for the growth of single- and multi-walled carbon nanotubes. Block copolymer templates are also used for the fabrication of nanocatalysts, mesoporous nanoparticles and film catalysts, gyroid-based bicontinuous catalysts, and hollow-fibre membrane catalysts. The block copolymers discussed in the review paper includes the following segments: poly(acrylic acid) (PAA), poly(2-vinyl pyridine) (P2VP), poly(4-vinyl pyridine) (P4VP), poly(ethylene glycole) (PEG), poly(ethylene oxide) (PEO), poly(propylene oxide) (PPO), poly(L-lactide) (PLLA), and poly(ferrocenyldimethylsilane) (PFS). The catalysts reconsidered are based on bimetallic alloy catalysts composed of PtAu, PtIr, and AuAg, Au–CeO_2_ strawberry-like and Janus nanoparticles, trimetallic mesoporous catalysts made of Cu, Ni, and Au precursors, and mesoporous Au–SiO_2_ yolk-shell nanoparticles [4].

In summary, these contributions show the importance of the development in the research area of block copolymer membranes and templates. The Special Issue includes specific approaches to reduce the amounts of polymer used in the fabrication of block copolymer membranes, challenges, and the need for further development in the field.
